# Clinician and staff experiences with frustrated patients during an electronic health record transition: a qualitative case study

**DOI:** 10.1186/s12913-024-10974-5

**Published:** 2024-04-26

**Authors:** Sherry L. Ball, Bo Kim, Sarah L. Cutrona, Brianne K. Molloy-Paolillo, Ellen Ahlness, Megan Moldestad, George Sayre, Seppo T. Rinne

**Affiliations:** 1 VA Northeast Ohio Healthcare System, 10701 East Blvd., Research Service 151, 44106 Cleveland, OH USA; 2 Center for Healthcare Organization and Implementation Research, VA Boston Healthcare System, Boston, MA USA; 3 Department of Psychiatry, Harvard Medical School, Boston, MA USA; 4 Center for Healthcare Organization and Implementation Research, VA Bedford Healthcare System, Bedford, MA USA; 5 Division of Health Informatics & Implementation Science, Department of Population and Quantitative Health Sciences, University of Massachusetts Chan Medical School, Worcester, MA USA; 6 Seattle-Denver Center of Innovation for Veteran-Centered and Value-Driven Care, VHA Puget Sound Health Care System, Seattle, WA USA; 7 University of Washington School of Public Health, Seattle, WA USA; 8 Geisel School of Medicine at Dartmouth, Hannover, NH USA

**Keywords:** EHR transition, Patient experience, Clinician experience, Qualitative analysis

## Abstract

**Background:**

Electronic health record (EHR) transitions are known to be highly disruptive, can drastically impact clinician and staff experiences, and may influence patients’ experiences using the electronic patient portal. Clinicians and staff can gain insights into patient experiences and be influenced by what they see and hear from patients. Through the lens of an emergency preparedness framework, we examined clinician and staff reactions to and perceptions of their patients’ experiences with the portal during an EHR transition at the Department of Veterans Affairs (VA).

**Methods:**

This qualitative case study was situated within a larger multi-methods evaluation of the EHR transition. We conducted a total of 122 interviews with 30 clinicians and staff across disciplines at the initial VA EHR transition site before, immediately after, and up to 12 months after go-live (September 2020-November 2021). Interview transcripts were coded using a priori and emergent codes. The coded text segments relevant to patient experience and clinician interactions with patients were extracted and analyzed to identify themes. For each theme, recommendations were defined based on each stage of an emergency preparedness framework (mitigate, prepare, respond, recover).

**Results:**

In post-go-live interviews participants expressed concerns about the reliability of communicating with their patients via secure messaging within the new EHR portal. Participants felt ill-equipped to field patients’ questions and frustrations navigating the new portal. Participants learned that patients experienced difficulties learning to use and accessing the portal; when unsuccessful, some had difficulties obtaining medication refills via the portal and used the call center as an alternative. However, long telephone wait times provoked patients to walk into the clinic for care, often frustrated and without an appointment. Patients needing increased in-person attention heightened participants’ daily workload and their concern for patients’ well-being. Recommendations for each theme fit within a stage of the emergency preparedness framework.

**Conclusions:**

Application of an emergency preparedness framework to EHR transitions could help address the concerns raised by the participants, (1) mitigating disruptions by identifying at-risk patients before the transition, (2) preparing end-users by disseminating patient-centered informational resources, (3) responding by building capacity for disrupted services, and (4) recovering by monitoring integrity of the new portal function.

## Background

Electronic health record (EHR) transitions present significant challenges for healthcare clinicians and staff. These transitions require adjustments in care delivery and may threaten care quality and value. It is critical that healthcare organizations undergoing these changes learn from others who have undergone similar transitions [1, 2]. However, the current literature lacks adequate guidance on navigating EHR transitions, especially as they relate to how clinicians and staff interact with patients [3].

Embedded within EHRs, patient portals facilitate complete, accurate, timely, and unambiguous exchange of information between patients and healthcare workers [4, 5]. These portals have become indispensable for completing routine out-of-office-visit tasks, such as medication refills, communicating laboratory results, and addressing patient questions [6]. In 2003, the VA launched their version of a patient portal, myHealtheVet [7] and by 2017 69% of Veterans enrolled in healthcare at the VA had registered to access the patient portal [8]. Similar to other electronic portals, this system allows Veterans to review test results, see upcoming appointments, and communicate privately and securely with their healthcare providers.

EHR transitions can introduce disruptions to patient portal communication that may compromise portal reliability, impacting patient and clinician satisfaction, patients’ active involvement in self-management, and ultimately health outcomes [9]. During an EHR transition, patients can expect reductions in access to care even when clinician capacity and IT support are increased. Patients will likely need for more assistance navigating the patient portal including and using the portal to communicate with their providers [10]. Staff must be prepared and understand how the changes in the EHR will affect patients and safeguards must be in place to monitor systems for potential risks to patient safety. Building the capacity to respond to emerging system glitches and identified changes must be included in any transition plan. Although portal disruptions are likely to occur when a new EHR is implemented, we know little about how these disruptions impact healthcare workers’ interactions and care delivery to patients [11, 12].

Due to an urgency to raise awareness and promote resolution of these patient portal issues,, we utilized existing data from the first EHR transition site for the Department of Veterans Affairs (VA)’s enterprise-wide transition. We focused on end users’ responses to the question “How Veterans were affected by the transition?”. We used qualitative methods to begin to understand how provider and patient interactions were affected during and by the EHR transition. We explored the impact of the EHR transition on patients through healthcare workers’ vicarious and direct experiences with patients. Due to the high level of disruption in care delivery we draw on insights from an emergency preparedness framework [13] to generate a set of recommendations to improve healthcare workers’ experiences during EHR transitions. The emergency preparedness framework includes 4 phases of an iterative cycle that include: (1) building capacity to mitigate issues, (2) preparing for the inevitable onset of issues, (3) responding to issues as they emerge, and (4) strategies to recover from any damage incurred.

## Methods

### Setting

In early 2020, the VA embarked on an EHR transition from a homegrown, legacy EHR system, developed by VA clinicians and used since the 1990s, to a new commercial system by the Oracle-Cerner Corporation. The primary objectives of this transition were to standardize care and improve interoperability between VA Medical Centers nationwide and the Department of Defense (DoD). Spanning over a decade, this transition plan is scheduled to roll out to all VA medical centers and outpatient clinics.

In this manuscript, we present data from the Mann-Grandstaff VA Medical Center in Spokane, WA, VA’s first EHR transition site. The study uses qualitative methods with clinician and staff interviews as part of a larger multi-method evaluation of the EHR transition. Our overarching goal is to identify and share recommendations to improve VA’s EHR transition efforts; rather than be guided by a theoretical framework our study design including the interview guides [14, 15] were based primarily on what was being experienced. An experienced team of ten qualitative methodologists and analysts conducted the study.

This evaluation was designated as non-research/quality improvement work by the VA Bedford Healthcare System Institutional Review Board deeming it exempt from needing an informed consent. Study materials, including interview guides with verbal consent procedures, were reviewed and approved by labor unions and by the VA Bedford Healthcare System Institutional Review Board; all methods were carried out in accordance with local and national VA guidelines and regulations.

Interview guides and an outline of the data collection plans were reviewed and approved by relevant national unions before beginning recruitment.

### Recruitment

Recruitment began in July 2020, before the first site implemented the new EHR. Prior to collecting data, we met with site leadership to get buy-in and support for the study, understand local context, determine how the site was approaching the transition, and to obtain the names of clinicians and staff for potential interviews. All potential participants were invited by email to participate in a one-hour voluntary interview conducted on Microsoft Teams® about their experiences with this transition; we used snowball sampling during interviews to expand the pool of interviewees. Verbal permission for audio recording of interviews was obtained immediately prior to the interview. Interview participants were informed that they could skip any questions, pause or stop the recording, and stop the interview at any time and were invited to ask questions before beginning the interview.

Most participants were interviewed at multiple timepoints; these included pre-implementation interviews, brief check-ins, and post-implementation interviews (Table 1). At the end of the pre-implementation interview, participants were invited to participate in 3–4 additional, shorter (15–20 min), check-in interviews where information about any changes in the transition process, context, or experience could be discussed. Most initial interviewees, in addition to three new participants, participated in post-implementation interviews (35–60 min; approximately 2–3 months and 10–12 months after the implementation) to reflect on the entire transition process.

**Table 1:** Interview participants

	Stakeholder engagement meetings	Pre-go-live interviews	Check-ins	2-months post-go-live interviews	1-year post-go-live interviews	Total
Clinicians^a,^ and clinical leaders	11	11	22	14	13	71
Nurses^b^	0	6	12	4	5	27
Allied health professionals^c^	0	4	13	5	2	24
Total	11	21	47	23	20	122

^a^Physicians, Clinical Pharmacists, Psychologists ^b^RNs and LPNs ^c^Medical assistants, phlebotomists, counselors, audiologists, physical therapist

### Data collection

Experienced qualitative interviewers included PhD trained qualitative methodologist and masters level qualitative analysts (JB, SB, AC, EK, MM, GS) conducted individual interviews with clinicians and staff, aligning to a semi-structured interview guide with follow-up probes using the participant’s words to elicit rich responses grounded in the data [16]. The guide was designed to inform ongoing efforts to improve the rollout of the new EHR. Six main categories were covered in our interview guides, including (1) attitudes toward the new software, (2) information communicated about the transition, (3) training and education, (4) resources, (5) prior experience with EHRs, and (6) prior experiences with EHR transitions. After piloting the interview guide with a clinician, initial interviews were completed between September and October 2020 and averaged ∼ 45 min in duration. Two-month and one-year post-implementation interview guides included an additional question, “Has the Cerner transition affected Vets?”; data presented here largely draw from responses to this question. Check-ins (October 2020– December 2020) took ∼ 15 min; two-month post-implementation interviews (December 2020– January 2021) and one-year post-implementation interviews (October 2020 - November 2021) took ∼ 45 min. Audio recordings of all interviews were professionally transcribed. To ensure consistency and relationship building, participants were scheduled with the same interviewer for the initial and subsequent interviews whenever feasible (i.e., check-ins and post-implementation interviews). Immediately following each interview, interviewers completed a debrief form where highlights and general reflections were noted.

Throughout the data collection process, interviewers met weekly with the entire qualitative team and the project principal investigators to discuss the recruitment process, interview guide development, and reflections on data collection. To provide timely feedback to leadership within the VA, a matrix analysis [17] was conducted concurrently with data collection using the following domains: training, roles, barriers, and facilitators. Based on these domains, the team developed categories and subcategories, which formed the foundation of an extensive codebook.

### Data analysis

All interviewers also coded the data. We used inductive and deductive content analysis [18]. Interview transcripts were coded in ATLAS.ti qualitative data analysis software (version 9). A priori codes and categories (based on the overall larger project aims and interview guide questions) and emergent codes and categories were developed to capture concepts that did not fit existing codes or categories [18]. Codes related to patient experience and clinician interactions with patients were extracted and analyzed using qualitative content analysis to identify themes [18]. Themes were organized according to their fit within the discrete stages of an emergency preparedness framework to generate recommendations for future rollout. In total, we examined data from 111 interviews with 24 VA clinicians and staff (excluding the initial 11 stakeholder meetings (from the 122 total interviews) that were primarily for stakeholder engagement). We focused on participants’ responses related to their experiences interacting with patients during the EHR transition.

## Results

Exemplar quotes primarily came from participants’ responses to the question, “Has the Cerner transition affected Vets?” and addressed issues stemming from use of the patient portal. This included both clinicians’ direct experiences with the portal and indirect experiences when they heard from patients about disruptions when using the portal. We identified four themes related to clinicians’ and staff members’ reported experiences: (1) stress associated with the unreliability of routine portal functions and inaccurate migrated information; (2) concern about patients’ ability to learn to use a new portal (especially older patients and special populations); (3) frustration with apparent inadequate dissemination of patient informational materials along with their own lack of time and resources to educate patients on use of the new portal; and (4) burden of additional tasks on top of their daily workload when patients needed increased in-person attention due to issues with the portal.

### Stress associated with the unreliability of routine portal functions and inaccurate migrated information

One participant described the portal changes as, “It’s our biggest stress, it’s the patients’ biggest stress… the vets are definitely frustrated; the clinicians are; so I would hope that would mean that behind the scenes somebody is working on it” (P5, check-in).

Participants expressed significant frustration when they encountered veterans who were suddenly unable to communicate with them using routine secure messaging. These experiences left them wondering whether messages sent to patients were received.

Those that use our secure messaging, which has now changed to My VA Health, or whatever it’s called, [have] difficulty navigating that. Some are able to get in and send the message. When we reply to them, they may or may not get the reply. Which I’ve actually asked one of our patients, ‘Did you get the reply that we took care of this?’ And he was like, ‘No, I did not (P11, 2-months post)

Participants learned that some patients were unable to send secure messages to their care team because the portal contained inaccurate or outdated appointment and primary site information.

I’ve heard people say that the appointments aren’t accurate in there… veterans who have said, ‘yeah, it shows I’m registered,’ and when they go into the new messaging system, it says they are part of a VA that they haven’t gone to in years, and that’s the only area they can message to, they can’t message to the [site] VA, even though that’s where they’ve actively being seen for a while now. (P20, 2-months post)

After the EHR transition, participants noted that obtaining medications through the portal, which was once a routine task, became unreliable. They expressed concern around patients’ ability to obtain their medications through the portal, primarily due to challenges with portal usability and incomplete migration of medication lists from the former to the new EHR.

I think it’s been negative, unfortunately. I try to stay optimistic when I talk to [patients], but they all seem to be all having continued difficulty with their medications, trying to properly reorder and get medications seems to still be a real hassle for them. (P17, one-year post)

…the medications, their med list just didn’t transfer over into that list [preventing their ability to refill their medications]. (P13, 2-months post)

### Concern about patients’ ability to learn to use a new portal

Clinicians and staff expressed concerns around veterans’ ability to access, learn, and navigate a new portal system. Clinicians noted that even veterans who were adept at using the prior electronic portal or other technologies also faced difficulties using the new portal.

They can’t figure out [the new portal], 99% of them that used to use our [old] portal, the electronic secure messaging or emailing between the team, they just can’t use [the new one]. It’s not functioning. (P13, one-year post)

Apparently, there’s a link they have to click on to make the new format work for them, and that’s been confusing for them. But I still am having a lot of them tell me, I had somebody recently, who’s very tech savvy, and he couldn’t figure it out, just how to message us. I know they’re still really struggling with that. (P5, 2-months post)

And it does seem like the My Vet [my VA Health, new portal], that used to be MyHealtheVet [prior portal], logging on and getting onto that still remains really challenging for a large number of veterans. Like they’re still just unable to do it. So, I do think that, I mean I want to say that there’s positive things, but really, I struggle (P17, one-year post)

Participants recognized difficulties with the new system and expressed empathy for the veterans struggling to access the portal.

I think that a lot of us, individually, that work here, I think we have more compassion for our veterans, because they’re coming in and they can’t even get onto their portal website. (P24, one-year post)

Participants acknowledged that learning a new system may be especially difficult for older veterans or those with less technology experience.

But, you know, veterans, the general population of them are older, in general. So, their technologic skills are limited, and they got used to a system and now they have to change to a new one. (P13, 2-months post)

So, for our more elderly veterans who barely turn on the computer, they’re not getting to this new portal. (P8, check in)

And you know, I do keep in mind that this is a group of people who aren’t always technologically advanced, so small things, when it’s not normal to them, stymie them.(P13, one-year post)

Concerns were heightened for veterans who were more dependent on the portal as a key element in their care due to specific challenges. One participant pointed out that there may be populations of patients with special circumstances who depend more heavily on the prior portal, MyHealtheVet.

I have veterans from [specific region], that’s the way they communicate. Hearing impaired people can’t hear on the phone, the robocall thing, it doesn’t work, so they use MyHealtheVet. Well, if that goes away, how is that being communicated to the veteran? Ok? (P18, Check-in)

### Frustration with inadequate dissemination of information to veterans about EHR transition and use of new portal

Participants were concerned about poor information dissemination to patients about how to access the new portal. During medical encounters, participants often heard from patients about their frustrations accessing the new portal. Participants noted that they could only give their patients a phone number to call for help using the new system but otherwise lacked the knowledge and the time to help them resolve new portal issues. Some clinicians specifically mentioned feeling ill-equipped to handle their patients’ needs for assistance with the new portal. These experiences exacerbated clinician stress during the transition.

Our veterans were using the MyHealtheVet messaging portal, and when our new system went up, it transitioned to My VA Health, but that wasn’t really communicated to the veterans very well. So, what happened was they would go into their MyHealtheVet like they had been doing for all of these years, to go in and request their medications, and when they pulled it up it’d show that they were assigned to a clinician in [a different state], that they have no active medications. Everything was just messed up. And they didn’t know why because there was no alert or notification that things would be changing. (P8, check in)

I field all-day frustration from the veterans. And I love my job, I’m not leaving here even as frustrated as I am, because I’m here for them, not to, I’m here to serve the veterans and I have to advocate for them, and I know it will get better, it can’t stay like this. But I constantly field their frustrations.… So, I give them the 1-800 number to a Cerner help desk that helps with that, and I’ve had multiple [instances of] feedback that it didn’t help. (P13, one-year post)

And [the patients are] frequently asking me things about their medication [within the portal], when, you know, I can’t help them with that. So, I have to send them back up to the front desk to try to figure out their medications. (P17, one-year post)

### Veteran frustration and the burden of additional tasks due to issues with the portal

Clinicians reported that veterans expressed frustration with alternatives to the portal, including long call center wait times. Some veterans chose to walk into the clinic without an appointment rather than wait on the phone. Clinicians noted an increase in walk-ins by frustrated veterans, which placed added workload on clinics that were not staffed to handle the increase in walk-ins.

It’s been kind of clunky also with trying to get that [new portal] transitioned. And then that’s created more walk-ins here, because one, the vets get frustrated with the phone part of it, and then MyHealtheVet (prior portal) not [working], so they end up walking [into the clinic without an appointment]. (P19, check-in)

In terms of messages, they can’t necessarily find the clinician they want to message. We had a veteran who came in recently who wanted to talk to their Rheumatologist, and it’s like, yeah, I typed in their name, and nothing came up. So, they have to try calling or coming in. (P20, 2-months post)

In summary, participants described the new patient portal as a source of stress for both themselves and their patients.

## Discussion

In addition to their own direct experience using a new EHR to communicate with their patients, clinicians and staff can be affected by perceptions of their patients’ experiences during an EHR transition [19]. At this first VA site to transition to the new EHR, clinicians and staff shared their concerns about their patients’ experiences using the portal. They were particularly troubled by unreliability of the secure messaging system and challenges patients faced learning to use the new system without proper instruction. Moreover, clinicians were alarmed to hear about patients having to make in-person visits– especially unplanned (i.e., walk in) ones– due to challenges with the new portal. Each of these issues needs to be addressed to ensure veteran satisfaction. However, the only solution participants could offer to frustrated patients was the telephone number to the help desk, leaving them with no clear knowledge of a solution strategy or a timeline for resolution of the issues.

**Fig. 1:**
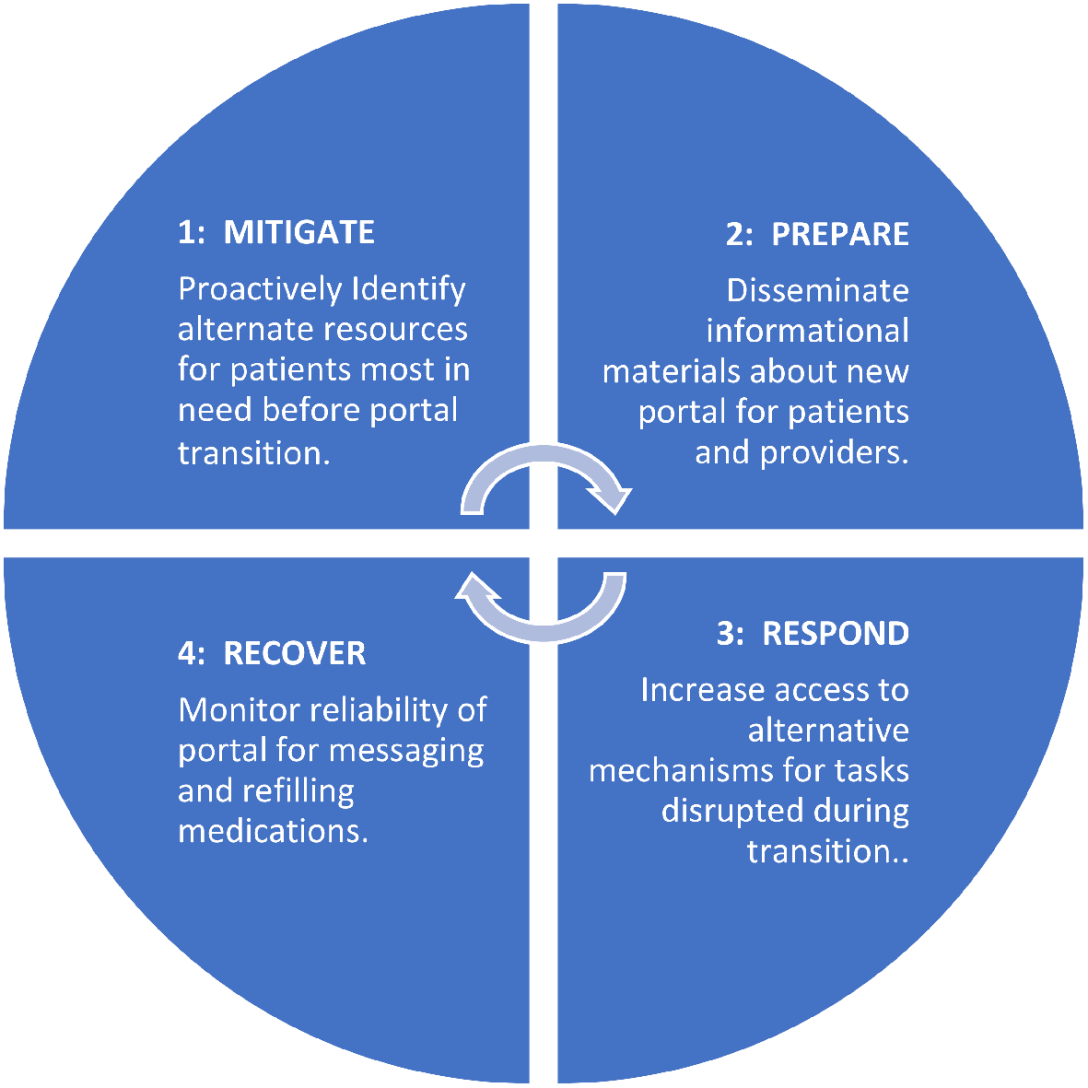
The emergency preparedness framework was applied. This framework includes 4 actions: (1) mitigate, (2) prepare, (3) respond, and (4) recover. These actions can be repeated. Recommendations for how each action (1–4) can be applied to a portal transition are included in each blue quadrant of the circle

We propose applying emergency preparedness actions to future EHR rollouts: mitigate, prepare, respond, and recover (Fig. 1) [13]. By applying these actions, patient portal disruptions may be alleviated and patients’ communication with their clinicians and access to care can be maintained. For example, issues stemming from a disruption in the portal may be **mitigated** by first identifying and understanding which patients typically use the portal and how they use it. Sites can use this information to **prepare** for the transition by disseminating instructional materials to staff and patients on how to access the new portal, targeting the most common and critical portal uses. Sites can **respond** to any expected and emerging portal disruptions by increasing access to alternative mechanisms for tasks disrupted by and typically completed within the portal. After the transition, **recovery** can begin by testing and demonstrating the accuracy and reliability of functions in the new portal. These actions directly address reported clinician concerns and can help maintain patient-clinician communication, and access to care.

### Mitigate

Sites could mitigate issues by first understanding which patients will be most affected by the transition, such as those who rely heavily on secure messaging. Reliable use of secure messaging within the VA facilitates positive patient-clinician relationships by providing a mechanism for efficient between-visit communication [20–23]. During the EHR transition, clinicians and staff became concerned about the well-being of patients from whom they weren’t receiving messages and those who depended on the portal to complete certain tasks. Since secure messaging is often initiated by patients to clinicians [23], clinicians will likely be unaware that messages are being missed. Understanding how and which patients currently use the portal and anticipating potential portal needs is a first step toward mitigating potential issues.

### Prepare

Despite efforts to inform Veterans of the EHR transition and patient portal [24] including information sent to a Veteran by email, direct mail, postings on VA websites, and a town hall, our findings agree with those of Fix and colleagues [10] and suggest that many Veterans were unprepared for the transition. Our findings suggest that end users heard that more is needed to improve the dissemination of knowledge about the transition and how to navigate the new patient portal to both VA employees and the patients they serve.

Preparations for the transition should prioritize providing VA clinicians and staff with updated information and resources on how to access and use the new portal [25]. VA clinicians deliver quality care to veterans and many VA employees are proud to serve the nation’s veterans and willing to go the extra mile to support their patients’ needs [26]. In this study, participants expressed feeling unprepared to assist or even respond to their patients’ questions and concerns about using the new portal. This unpreparedness contributed to increased clinician and staff stress, as they felt ill-equipped to help their patients with portal issues. Such experiences can negatively affect the patient-clinician relationship. Preparing clinicians and patients about an upcoming transition, including technical support for clinicians and patients, may help minimize these potential issues [10, 27]. Specialized training about an impending transition, along with detailed instructions on how to gain access to the new system, and a dedicated portal helpline may be necessary to help patients better navigate the transition [23, 28].

### Respond

In addition to a dedicated helpline, our recommendations include responding to potential changes in needed veteran services during the transition. In our study, participants observed more veteran walk-ins due to challenges with the patient portal. Health systems need to anticipate and address this demand by expanding access to in-person services and fortifying other communication channels. For example, sites could use nurses to staff a walk-in clinic to handle increases in walk-in traffic and increase call center capacity to handle increases in telephone calls [29]. Increased use of walk-in clinics have received heightened attention as a promising strategy for meeting healthcare demands during the COVID-19 pandemic [30] and can potentially be adapted for meeting care-related needs during an EHR transition. These strategies can fill a gap in communication between clinicians and their patients while patients are learning to access and navigate a new electronic portal.

### Recover

Finally, there is a need for a recovery mechanism to restore confidence in the reliability of the EHR and the well-being of clinicians and staff. Healthcare workers are experiencing unprecedented levels of stress [31]. A plan must be in place to improve and monitor the accuracy of data migrated, populated, and processed within the new system [2]. Knowing that portal function is monitored could help ease clinician and staff concerns and mitigate stress related to the transition.

## Limitations

This study has several limitations. First, data collection relied on voluntary participation, which may introduce self-selection response bias. Second, this work was completed at one VA medical center that was the first site in the larger enterprise-wide transition, and experiences at other VAs or healthcare systems might differ substantially. Third, we did not interview veterans and relied entirely on secondhand accounts of patient experiences with the patient portal. Future research should include interviews with veterans during the transition and compare veteran and VA employee experiences.

## Conclusion

Despite a current delay in the deployment of the new EHR at additional VA medical centers, findings from this study offer timely lessons that can ensure clinicians and staff are equipped to navigate challenges during the transition. The strategies presented in this paper could help maintain patient-clinician communication and improve veteran experience. Guided by the emergency preparedness framework, recommended strategies to address issues presented here include alerting those patients most affected by the EHR transition, being prepared to address patients’ concerns, increasing staffing for the help desk and walk-in care clinics, and monitoring the accuracy and reliability of the portal to provide assurance to healthcare workers that patients’ needs are being met. These strategies can inform change management at other VA medical centers that will soon undergo EHR transition and may have implications for other healthcare systems undergoing patient portal changes. Further work is needed to directly examine the perspectives of veterans using the portals, as well as the perspectives of both staff and patients in the growing number of healthcare systems beyond VA that are preparing for an EHR-to-EHR transition.

## Data Availability

Deidentified data analyzed for this study are available from the corresponding author on reasonable request.

## Declarations

### Prior presentations

Ball S, Kim B, Moldestad M, Molloy-Paolillo B, Cubanski L, Cutrona S, Sayre G, and Rinne S. (2022, June). Electronic Health Record Transition: Providers’ Experiences with Frustrated Patients. Poster presentation at the 2022 AcademyHealth Annual Research Meeting. June 2022.

## Abbreviations

HER: Electronic health record
VA: Department of Veterans Affairs
VAMCs: VA Medical Centers
DoD: Department of Defense
